# Dermatology

**Published:** 1908-02-29

**Authors:** 


### Dermatology.

Among the cases at the Wednesday Skin Clinic at the Prince of Wales's General Hospital, Tottenham, the following presented several features of interest:—

### Hirsuties with Mental Disorder.

A rather stout woman, aged forty, complained of a growth of hair upon her chin for the last nine months. As she was the subject of chronic alcoholism, and possessed of a loud and raucous voice, her masculine aspect produced a mild sensation when she entered the consulting-room. She had quite a presentable beard, reaching down the neck as far as the upper margin of the larynx, but there was no hair upon other parts of the face. She was unmarried, and her menstruation was now very irregular. Her conversation was very incoherent and rambling, and it was soon evident that she was suffering from mental delusions. The connection between facial hypertrichosis and menstrual troubles was known to Hippocrates, and it is not uncommon to see some slight degree of hirsuties in women at or about the climacteric period. It is also met with among the insane. The question of treatment is a difficult one. In the present case to have recommended shaving would have been most ill-advised, and to have given her depilatories to use herself would have been little better. It was decided, therefore, to apply pure liquor potassæ to the affected parts by means of a compress then and there, which was accordingly done, the alkali being allowed to act for ten minutes. The surface was then gently scraped, not shaved, some of the hairs coming away, the parts being then bathed with a very dilute solution of acetic acid. A little zinc oxide was then applied, and the patient sent home.

### Alopecia after an Acute Illness.

A boy of ten came with extensive patchy baldness, which was stated to have first appeared a month after he had had rheumatic fever six months ago. His mother, with the assistance of the local chemist, had been treating it assiduously for "ringworm." He was said to be a very nervous lad, and in infancy he cut his teeth "with fits." The alopecia was of the mixed patchy and band type, the largest area being over the left parietal region, while a wide belt extended backwards from this part towards the occiput to meet with a smaller patch upon the right side of the head. Close examination revealed a few "point of exclamation" hairs, but no stumps, such as would be seen in tinea tonsurans. The dentition was most irregular, the central incisor teeth being especially crowded together and out of line, but there was no caries. The eyesight was said to be normal, though this was not tested at the hospital. True alopecia areata, or "area Celsi," may, of course, appear soon after or during an illness as it may at any other time, but it is rather more common to meet with a general thinning of the hair, the loss of which may prove quite alarming, owing to defective nutrition of the scalp itself. The question as to whether the whole hair should be cropped short during an acute illness, such as scarlet fever, is one that has been much debated, one modern authority, at least, regarding the practice as "absolutely unnecessary, barbarous, and without common sense to support it." On the other hand, many of the older dermatologists believed that the habit was not only justifiable but really beneficial, tending to promote hair growth. Short hair is more comfortable in bed, and it is both easier to keep clean and to disinfect afterwards, than when long tresses are worn. The prognosis in these cases is, happily, good. A useful remedy for local application is chloral hydrate in the strength of five grains to the ounce of lanoline. A little red oxide of mercury may also be incorporated with the ointment with advantage. The rubbing must be undertaken cautiously at first, and this is a pretty safe rule for all cases of alopecia, be they ever so stubborn, for one can never tell how a given scalp is going to react to stimulants when locally applied. The spirituous lotion of cantharidis may be employed at the same time, and it will also be generally necessary to give some internal tonic, such as iron or strychnine.

